# Unmasking culprits: novel analysis identifies complement factors as potential therapeutic targets to mitigate inflammation during children's heart surgery

**DOI:** 10.1186/s40001-024-02156-0

**Published:** 2024-12-19

**Authors:** Joel Bierer, Roger Stanzel, Mark Henderson, John Sapp, Pantelis Andreou, Jean S. Marshall, David Horne

**Affiliations:** 1 Division of Cardiac Surgery, IWK Children’s Heart CentreDivision of Cardiac Surgery, Dalhousie University, Halifax, Canada; 2 Department of Clinical Perfusion, Nova Scotia Health Authority, Halifax, Canada; 3 Division of Cardiology, Dalhousie University, Halifax, Canada; 4 Department of Community Health and Epidemiology, Dalhousie University, Halifax, Canada; 5 Department of Microbiology and Immunology, Dalhousie University, Halifax, Canada

**Keywords:** Pediatric cardiac surgery, Congenital heart disease, Cardiopulmonary bypass, Complement, Inflammation

## Abstract

**Background:**

Cardiopulmonary bypass (CPB) causes systemic inflammation during pediatric cardiac surgery, which can contribute to post-operative organ dysfunction and prolonged recovery. This study aims to identify key inflammatory mediators related to this clinically significant immunologic response.

**Methods:**

Pediatric patients were enrolled in a single-arm prospective clinical study (NCT05154864) and received standard cardiac operation, CPB and subzero-balance ultrafiltration. Arterial samples were taken before CPB initiation and immediately after weaning, and concentrations of 33 inflammatory mediators were assayed. A principal component analysis with hierarchical clustering (PCA-HCPC) included inflammatory mediator concentrations measured at the end of CPB, validated peak post-operative clinical scores, ventilation time and intensive care length of stay. Mahalanobis distance assessed statistical differences between clusters. Spearman’s correlation described the linear relationship between mediator concentrations at the end of CPB and intensive care length of stay. Results are median (IQR).

**Results:**

Forty consecutive patients were enrolled; the majority were male (58%), age of 7.3 (1.7–39.0) months and weight of 6.7 (4.6–14.9) kg. The PCA-HCPC revealed activated complement factors along with all peak clinical scores and prolonged intensive care requirements in the same cluster. Cytokine, chemokine, and leukocyte adhesion molecule concentrations were found in two other distinct clusters (Mahalanobis distance = 16.5; *p* = 0.004 and Mahalanobis distance = 17.4; *p* = 5.8 × 10^–4^). Mediator concentrations of C2 (Rho = 0.50; *p* = 0.001), C3 (Rho = 0.58; *p* = 1.1 × 10^–4^), C3b (Rho = 0.47; *p* = 0.002), C5 (Rho = 0.48; *p* = 0.002) and C5a (Rho = 0.63; 1.7 × 10^–5^) showed linear correlations with intensive care unit length of stay.

**Conclusions:**

Activated complement factors, but not pro-inflammatory cytokines or chemokines, were most related to cardiopulmonary dysfunction and prolonged recovery in this novel analysis. Investigation of therapies that inhibit complement to dampen CPB-associated inflammation and enhance recovery after pediatric cardiac surgery is warranted.

*Trial Registration* ClinicalTrials.gov, NCT05154864

**Supplementary Information:**

The online version contains supplementary material available at 10.1186/s40001-024-02156-0.

## Introduction

Cardiopulmonary bypass (CPB) during pediatric heart surgery is associated with systemic inflammation [1, 2]. The non-endothelialized circuit concomitantly activates the alternative complement pathway, coagulation system, the contact system and the inflammatory response is further stimulated by cellular injury via ischemia–reperfusion, surgical trauma, and hypothermia [2, 3]. Potent anaphylatoxins C3a and C5a are produced and stimulate neutrophil activation and release of pro-inflammatory cytokines, which directly facilitate endothelial leak syndrome, neutrophil recruitment, tissue edema, and subsequent injury that contribute to end-organ dysfunction in the post-operative period [4, 5]. Clinically, these phenomena can present as vasomotor dysfunction, low cardiac output syndrome, respiratory failure and renal insufficiency, which are associated with longer intensive care unit length of stay (ICU LOS), higher morbidity and mortality [2, 5, 6].

Unfortunately, there are still no proven immunomodulatory therapies that dampen CPB-associated inflammation and enhance recovery after pediatric cardiac surgery. Both prophylactic steroids, via inhibition of nuclear factor kappa beta (NF-κβ), and intra-operative nitric oxide have been assessed by randomized trials without consistent evidence of clinical anti-inflammatory benefit [7–9]. This suggests more research is needed to identify the key mediators or inflammatory systems driving the post-operative clinical syndrome of CPB-associated inflammation, thereby marking possible therapeutic targets.

Ultrafiltration during CPB is a potential candidate to dampen inflammation and enhance recovery after pediatric cardiac surgery as it directly extracts activated complement components and pro-inflammatory cytokines [1, 10]. There are a variety of ultrafiltration protocols that can be categorized as continuous forms of ultrafiltration, used throughout the entire CPB time, or non-continuous forms of ultrafiltration, which are used only after the patient is weaned from CPB, such as modified ultrafiltration (MUF) [1, 11]. Conceptually, continuous forms of ultrafiltration offer a more advantageous therapeutic profile as it extracts noxious inflammatory mediators through the entire CPB exposure, rather than a few minutes at the end of CPB [1, 12]. To this end, we have developed a combination method that includes subzero-balance ultrafiltration (SBUF) during the CPB time followed by simple modified ultrafiltration (SMUF) at CPB cessation; together termed SBUF–SMUF [12]. The purpose of this prospective clinical study is to conduct a comprehensive exploratory analysis to identify which inflammatory mediators—complement factors, cytokines, chemokines, and leukocyte adhesion molecules—are related to end-organ dysfunction and prolonged recovery after pediatric cardiac surgery.

## Methods

This single-center and single-arm prospective clinical trial (NCT05154864 on ClinicalTrials.gov) investigates the relationship between circulating inflammatory mediators and post-operative clinical outcomes in pediatric patients undergoing cardiac surgery with CPB and SBUF–SMUF. Written informed consent was obtained from substitute decision-makers for all participants under a protocol approved by the IWK Health Centre Research Ethics Board (#1024869) on November 21, 2019. Patients were enrolled between August 2020 and June 2021. A previous publication examined the efficiency of inflammatory mediator extraction by ultrafiltration in a subset of this patient group; the results here presented are unique and not duplicated [10].

### Study participants

Patients weighing less than 30 kg undergoing congenital cardiac surgery with CPB were prospectively enrolled. Exclusion criteria included: absence of written consent, known severe hematologic abnormality, genetic syndrome with severe multi-organ involvement, immunodeficiency syndrome and severe liver disease. Participating patients were prospectively followed throughout their cardiac procedure with standard CPB, SBUF–SMUF, anesthesia and routine institutional post-operative management. Steroids were administered at the discretion of the anesthetist according to local standards; neonates received prophylactic methylprednisolone and older children received anti-emetic doses of dexamethasone.

### CPB and SBUF–SMUF technique

A technical overview of our pediatric CPB with SBUF–SMUF method has been previously reported [12]. A *Liva Nova S5™* CPB System with phosphorylcholine coating (48-40-00, London, UK), *Terumo FX05* or *FX15* oxygenators (1CX*FX05RE/1CX*FX15E, Tokyo, Japan) and *Terumo Capiox*^*®*^ Hemoconcentrator HCO5 (1CX*HC05S, Tokyo, Japan) were used. Per the manufacturer, this ultrafiltration device has a sieving Coefficient of 0.2% for albumin, which has a molecular weight of 66 kDa. Sanguineous CPB prime was used for patients < 10 kg, while a crystalloid prime with retrograde autologous prime was used for those > 10 kg. Buffered ultrafiltration of the CPB circuit prime (BUF) was used to normalize metabolic abnormalities in sanguineous prime before CPB initiation [13]. Once full flow CPB was reached, SBUF was initiated for the remainder of CPB in a post-pump pre-oxygenator veno-venous configuration; 30 ml/kg/h of effluent was removed while 25 ml/kg/h of a physiologic solution was infused to target a net balance of − 5 ml/kg/h [12]. Precise volumes of effluent removal and replacement are facilitated by *Braun Infusomat*^*®*^* Space* pumps (8710351U, Frankfurt, Germany). Cardioplegia and surgical field irrigation volumes were removed via the hemoconcentrator in addition. SBUF was paused during deep hypothermic circulatory arrest (DHCA). Just prior to weaning the patient from CPB, SBUF was deactivated. Immediately after separation from bypass, SMUF was initiated in a veno-arterial fashion with an endpoint target of venous reservoir depletion or reaching goal hematocrit of 40%. For both SBUF and SMUF, 5% of the calculated cardiac output was shunted to the hemoconcentrator.

### Data collection

Baseline demographic and clinical information was recorded from the medical record and follow-up continued until discharge from the pediatric ICU. Intraoperative data including CPB time, cross-clamp time, type of CPB prime, ultrafiltration volumes and fluid balance, were collected from the perfusion and anesthesia records. Arterial blood (1 ml) was drawn post-sternotomy but prior to CPB initiation (Pre-CPB), and another 1 ml of arterial blood was drawn at the end of CPB and SMUF (End-CPB) for inflammatory mediator analysis.

Post-operative clinical outcomes included 30-day mortality, mechanical circulatory support requirement, acute kidney injury defined by the pediatric Kidney Disease Improving Global Outcome (KDIGO) criteria, delayed chest closure, ventilation time and ICU LOS (pre-specified standard criteria shown in Supplement Table A). Four validated pediatric intensive care clinical scores—vasoactive-ventilation-renal score (VVR), vasoactive-inotrope score (VIS), ventilation index (VI) and oxygenation index (OI)—were used to describe cardiopulmonary, renal and vasomotor function after anesthesia induction but before CPB initiation (Pre-CPB), immediately after CPB and SMUF (End-CPB), at ICU Admission, and 12-, 24-, 48-, 72-, 96-, 120-h Post-CPB [14–17]. All four clinical scores behave similarly, with an increasing score indicating a higher level of organ dysfunction and medical instability. A score of 0 indicates no requirement for inotropic or mechanical ventilatory support. The peak VVR, VIS, VI, and OI were the highest individual clinical score recorded in the ICU.

### Immunoanalysis

Arterial blood samples were collected in EDTA tubes, centrifuged for 10 min (0.5 × gravity), and the resulting plasma was extracted. The plasma underwent a second centrifugation for 20 min (2.5 × gravity) to yield a platelet-free plasma which was aliquoted, flash-frozen in liquid nitrogen and stored at – 80 °C. *Luminex* immunoanalysis of a panel of relevant mediators was completed with a *Bio-Rad Bio-Plex*^*®*^* 200* System (Hercules, United States). Thirty-three pre-specified human inflammatory factors were analyzed using multiple analysis kits including: *ThermoFisher* C3a Simplex Kit (EPX010-12282-901, Waltham, United States), *Millipore Sigma* Human Complement Magnetic Bead Panel 1 (HCMP1MAG-19K-05, Burlington, United States), *Millipore Sigma* Human Complement Magnetic Bead Panel 2 (HCMP2MAG-19K-06, Burlington, United States), *BioTechne R&D Systems* Human XL Cytokine Luminex Performance Panel (FCSTM18-21, Minneapolis, United States), *BioTechne R&D Systems* Human Magnetic Luminex Assay (LXSAHM-05, Minneapolis, United States) and *BioTechne R&D Systems* Human Magnetic Luminex Assay (LXSAHM-01, Minneapolis, United States). Bio-Rad Bio-Plex^®^ ManagerTM Software 6.2 (Hercules, United States) was used to complete the data acquisition and used Logistic—5PL regression for all analytes. All assays were conducted according to the manufacturer’s instructions.

### Statistical analysis

Categorical variables are reported as numbers (%), and continuous variables are presented as median (interquartile range). The difference in inflammatory mediator concentration between Pre-CPB and End-CPB was assessed in a paired fashion with Wilcoxon signed-rank test, and the median difference (MD) [95% confidence interval] was calculated by the exact permutation probability technique.  Statistical significance for pairwise comparison of mediator concentrations at Pre- and End-CPB was corrected by Bonferroni method with an α = 0.002. The magnitude of mediator concentration change at End-CPB relative to Pre-CPB baseline was expressed as a median fold change ([End-CPB] — [Pre-CPB]/[Pre-CPB]) with [95% confidence interval] estimated by 1000 non-parametric bootstrap samples. Evolution of clinical scores throughout the time series was also assessed in a pairwise fashion by Wilcoxon signed-rank test.

A principal component analysis (PCA) with hierarchical clustering on principal components (HCPC) was conducted on R with “FactoMineR” [18, 19]. PCA transforms large multi-variable datasets into reduced forms by creating principal components which contain information and removing excessive variability of uncorrelated variables [19]. HCPC builds upon the refined principal components and uses Ward’s method to aggregate groups of variables with minimal variance [18, 19]. Forty-two variables were included in the exploratory analysis, including concentrations of all 33 inflammatory mediators and lactate measured at End-CPB, CPB time, cross-clamp time, ICU LOS, ventilation time, peak VVR, peak VIS, peak VI and peak OI. Multiple imputations by “missMDA” on R for the principal component analysis was used for six missing datapoints [20]. Overall, this represents an insignificant amount of imputed data (6/1680) = 0.4%. Mahalanobis distance was calculated to describe the differences between clusters, through all dimensions of the principal component analysis, and statistically evaluated by the Hotelling T^2^ test, F Statistic, and corresponding p-value [21].

Mediators that had showed dynamic increases throughout CPB or clustered with post-operative clinical variables in the PCA-HCPC were assessed with Spearman’s linear correlation between the End-CPB mediator concentration with ICU LOS and Peak VVR as two robust surrogates of critical care requirements and post-operative recovery [14, 22]. Statistical significance for the linear correlations was again adjusted by Bonferroni method for this correlation analyses to consider multiple comparisons with α = 0.004.

## Results

### Patient population

During the study period, 50 consecutive pediatric patients were assessed for trial enrollment. There were 40 who consented and completed the protocol, while 10 patients were not enrolled due to exclusion factors: a genetic syndrome with severe multi-organ abnormalities (3), weight over 30 kg (3), unavailable research coordinator (2) and patient refusal to participate (1). The baseline characteristics of the group are summarized in Table 1. The majority of patients were male (58%), less than one year old (55%) and had a variety of cardiac pathologies with the Society of Thoracic Surgeons-European Association for Cardio-Thoracic Surgery (STAT) risk scores between 1 and 4.

**Table 1:** Patient demographics (*n* = 40)

	No. (%), median (IQR)
Sex	
Male	23 (58%)
Female	17 (42%)
Age (months)	7.3 (1.7–39.0)
Neonate (< 30 days)	10 (25%)
Infant (30 days–1 year)	12 (30%)
Child (> 1 year)	18 (45%)
Weight (kg)	6.7 (4.6–14.9)
Body surface area (m^2^)	0.35 (0.27–0.64)
Single ventricle	6 (15%)
STAT score	
1	15 (37%)
2	10 (25%)
3	2 (5%)
4	13 (33%)
Congenital heart pathology	
Atrial septal defect	4 (10%)
Ventricular septal defect	8 (20%)
Sub-aortic stenosis	3 (9%)
Right ventricular outflow tract obstruction	2 (5%)
Tetralogy of Fallot	2 (5%)
Double outlet right ventricle	1 (2%)
Incomplete atrioventricular septal defect	2 (5%)
Complete atrioventricular septal defect	1 (2%)
Total anomalous pulmonary venous return	4 (10%)
d-Transposition of the great arteries	4 (10%)
Aorto-pulmonary window and interrupted arch	1 (2%)
Truncus arteriosus	1 (2%)
Aortic arch hypoplasia	1 (2%)
Single ventricle—central shunt	1 (2%)
Single ventricle—bidirectional Glenn	2 (5%)
Single ventricle—Fontan	3 (9%)

*STAT* Society of Thoracic Surgeons-European Association for Cardio-Thoracic Surgery

### Intraoperative clinical and immunologic data

All patients underwent planned cardiac operations; the intra-operative data are summarized in Table 2. Fourteen (35%) patients received intravenous steroids at anesthesia induction with a median prednisone equivalent of 12 (11–21) mg/kg. SBUF was used in all 40 (100%) patients, while SMUF was used in 37 (93%) patients. Consistent with the volume balance target of SBUF–SMUF, most patients had a negative volume balance during CPB of -11 (- 25 – - 5) ml/kg. There were no intra-operative perfusion- or ultrafiltration-related complications. One patient was transitioned from CPB to central extracorporeal membrane oxygenation due to post-cardiotomy low cardiac output syndrome before transfer to the ICU.

**Table 2:** Clinical data (*n* = 40)

	No. (%), Median (IQR)
Intra-operative data	
Steroid administration (count, prednisone-eq mg/kg)	14 (35%), 12 (11–21)
CPB time (count, minutes)	40 (100%), 170 (130–260)
Cross clamp time (count, minutes)	36 (90%), 93 (78–128)
Deep hypothermic circulatory arrest (count, minutes)	8 (20%), 35 (25–60)
Lowest temperature (˚C)	30.0 (26.0–32.0)
Sanguineous prime	26 (65%)
SBUF (count, effluent ml/kg)	40 (100%), 155 (100–185)
SMUF (count, effluent ml/kg)	37 (93%), 17 (10–35)
Total ultrafiltration effluent volume (ml/kg)	189 (109–222)
Urine output during CPB (ml/kg)	20 (7 to 34)
Cardiopulmonary bypass volume balance (ml/kg)	-11 ( -25 – -5)
Anesthesia volume balance (ml/kg)	14 ( -3–40)
Post-operative clinical outcomes	
Mortality (30-day)	0
Mechanical circulatory support	1 (2%)
Acute kidney injury	3 (8%)
Grade 1	3 (8%)
Grade 2	0
Grade 3	0
Delayed chest closure	7 (18%)
Ventilation time (hours)	19 (0–70)
ICU length of stay (hours)	62 (24–95)
Clinical scores	
Peak VVR	24.3 (6.5–31.3)
Peak VIS	7.0 (5.0–14.5)
Peak VI	16.0 (0.0–19.3)
Peak OI	3.0 (0.0–4.8)

*CPB* cardiopulmonary bypass, *ICU* intensive care unit, *OI* oxygenation index, *SBUF* subzero-balance ultrafiltration, *SMUF* simple modified ultrafiltration, *VI* ventilation index, *VIS* vasoactive-inotrope scores, *VVR* ventilation-vasoactive-renal score

**Fig. 1:**
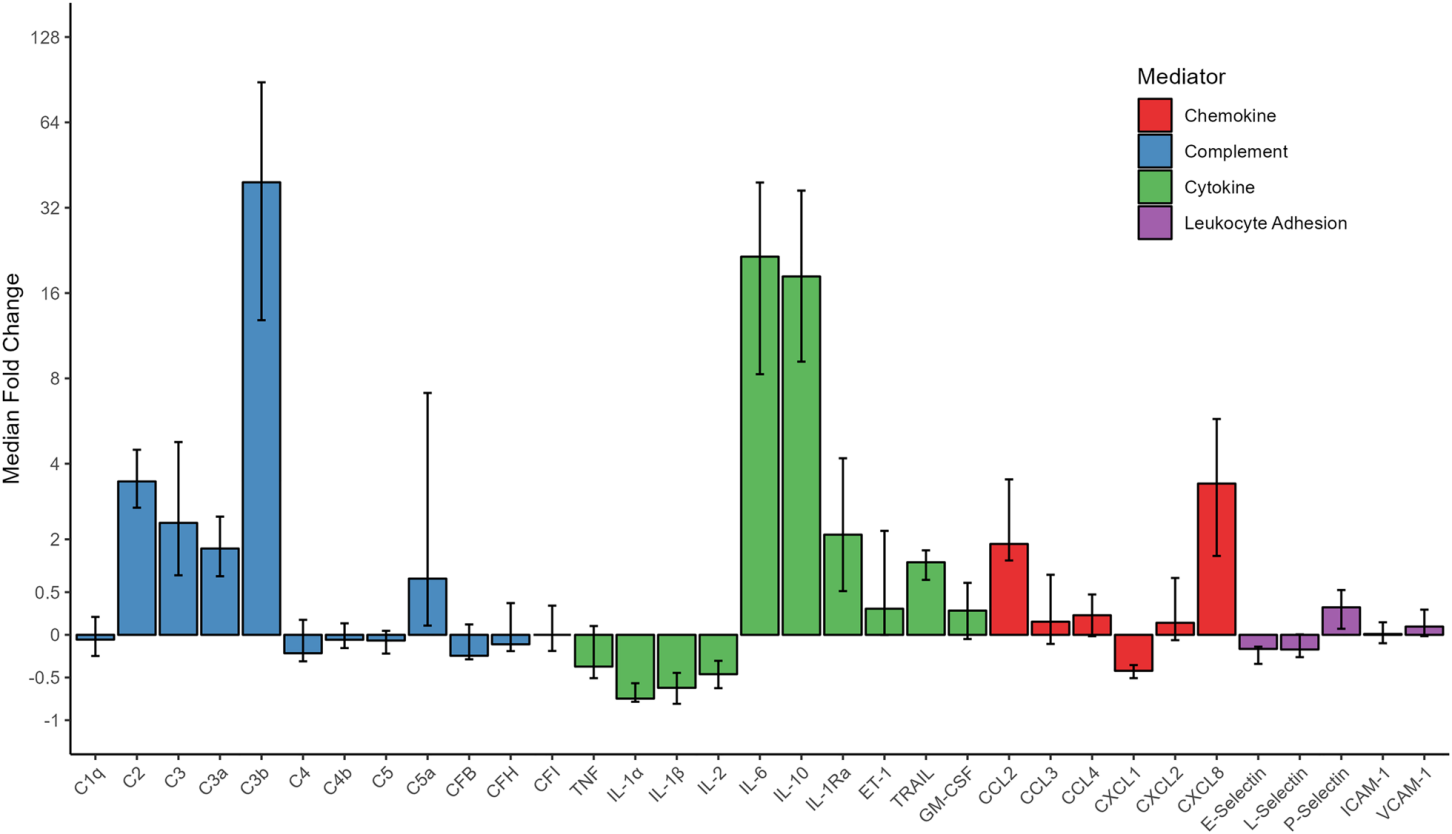
Mediator dynamic changes during CPB. Median fold change represents the increase or decrease of the mediator concentration between the Pre-CPB and End-CPB measurements with 95% confidence intervals. C: complement; CF: complement factor; CCL: CC chemokine ligand; CXCL: CXC chemokine ligand; ET1: endothelin-1; GM-CSF: granulocyte–macrophage colony-stimulating factor; ICAM: intracellular adhesion molecule; IL: interleukin; TNF: tumor necrosis factor; TRAIL: tumor necrosis factor-related apoptosis-inducing ligand; VCAM: vascular cell adhesion molecule

The changes in inflammatory mediator concentrations from Pre-CPB to End-CPB were variable across the complement factors, cytokines, chemokines and leukocyte adhesion molecules. Table 3 describes mediator behaviors with pairwise comparison between Pre-CPB baseline and End-CPB, while Fig. 1 illustrates the median fold change for each mediator. The complement components C2 (3.5x median fold increase), C3 (2.4x), C3a (1.8x), C3b (39.3x), and C5a (1.2x), along with pro-inflammatory cytokine IL-6 (21.5x) and chemokine CXCL8 (3.4x), showed substantial elevation throughout the CPB time. Furthermore, the anti-inflammatory regulators IL-1Ra (2.1x) and IL-10 (18.3x) were markedly increased by the end of the CPB exposure. In contrast, important pro-inflammatory cytokines such IL-1α (-0.8 median fold decrease) and IL-1β (-0.6x) were reduced relative baseline while TNF did not change.

**Table 3:** Inflammatory mediator concentrations during cardiopulmonary bypass (*n* = 40)

Inflammatory mediator	Pre-CPB Plasma (IQR)	End-CPB Plasma (IQR)	Median difference [95% CI]	*p*-value	Change
C1q (µg/ml)	47.2 (33.9–72.8)	53.5 (28.7–84.3)	2.6 [ -10.5–24.1]	0.71	↔
C2 (µg/ml)	0.4 (0.3–0.6)	2.0 (1.2–3.0)	1.7 [1.3–2.1]	1.8 × 10^–12^	**↑**
C3 (µg/ml)	15.3 (9.8–22.9)	55.0 (24.8–113.5)	53.1 [35.6–76.2]	4.5 × 10^–11^	**↑**
C3a (ng/ml)	17.6 (9.0–26.4)	52.0 (35.0–60.0)	30.0 [24.1–36.5]	1.0 × 10^–7^	**↑**
C3b (µg/ml)	6.6 (1.5–27.7)	290.0 (164.7–519.5)	324.8 [230.8–472.7]	2.3 × 10^–9^	**↑↑↑**
C4 (µg/ml)	154.8(105.5–218.2)	155.6 (91.1–214.9)	-8.9 [ -42.7–33.8]	0.74	↔
C4b (µg/ml)	12.2 (10.5–13.8)	11.6 (10.0–13.4)	-0.5 [-1.7–0.6]	0.40	↔
C5 (µg/ml)	12.8 (10.0–15.0)	11.4 (8.7–17.1)	-0.6 [-2.1–1.1]	0.40	↔
C5a (pg/ml)	20.1 (7.8–73.4)	109.9 (24.0–268.9)	106.4 [56.2–192.6]	2.2 × 10^–5^	**↑↑**
CFB (µg/ml)	150.9 (98.8–185.7)	136.6 (77.3–182.1)	-9.5 [-36.2–27.0]	0.64	↔
CFH (µg/ml)	176.3 (128.7–261.9)	193.6 (111.5–313.7)	10.4 [-31.9–74.1]	0.68	↔
CFI (µg/ml)	19.6 (15.2–28.2)	21.9 (17.9–27.9)	-1.3 [-8.7–4.4]	0.66	↔
ET-1 (pg/ml)	1.2 (0.4–3.3)	1.8 (0.9–5.8)	2.7 [0.8–5.6]	0.01	**↑**
TNF (pg/ml)	28.7 (21.6–37.3)	19.0 (10.7–74.9)	7.3 [− 11.7–67.5]	0.74	↔
IL-1α (pg/ml)	57.7 (42.6–64.9)	17.7 (7.3–31.4)	-38.0 [-44.8–-31.3]	5.9 × 10^–8^	↓
IL-1β (pg/ml)	7.9 (5.3–9.7)	2.9 (1.0–4.6)	-5.1 [-6.3– -3.5]	2.7 × 10^–7^	↓
IL-2 (pg/ml)	12.4 (8.3–15.5)	5.7 (4.3–9.8)	-5.1 [-6.9–-3.0]	2.5 × 10^–4^	↓
IL-6 (pg/ml)	10.2 (4.6–23.4)	245.5 (99.8–754.2)	375.1 [179.2–563.8]	1.8 × 10^–12^	**↑↑↑**
IL-10 (pg/ml)	77.3 (37.6–113.0)	1438 (264–4193)	2125 [1244–3915]	3.6 × 10^–12^	**↑↑↑**
IL-1Ra (ng/ml)	0.4 (0.3–0.6)	1.9 (0.9–6.3)	1.7 [1.0–4.9]	5.3 × 10^–7^	**↑**
TRAIL (pg/ml)	165.1 (124.0–211.7)	319.5 (224.7–460.2)	176.9 [127.6–229.1]	3.9 × 10^–7^	**↑**
GM-CSF (pg/ml)	19.4 (11.7–26.6)	24.0 (17.4–28.2)	5.8 [0.6–11.4]	0.03	**↑**
CCL2 (pg/ml)	202.0 (166.8–261.9)	601.9 (340.7–1352)	611.0 [325.9–1003.0]	8.1 × 10^–10^	**↑**
CCL3 (pg/ml)	31.5 (26.5–37.4)	33.5 (27.2–104.7)	19.1 [2.1–118.3]	0.02	**↑**
CCL4 (pg/ml)	519.3 (466.7–644.1)	602.1 (471.0–946.7)	155.2 [26.6–425.6]	0.01	**↑**
CXCL1 (pg/ml)	171.4 (134.8–219.7)	96.8 (66.9–165.7)	-67.6 [-91.0– -40.0]	4.3 × 10^–4^	↓
CXCL2 (ng/ml)	2.0 (1.1–2.7)	2.3 (1.5–3.4)	0.4 [-0.2–1.2]	0.17	↔
CXCL8 (pg/ml)	11.1 (8.6–17.6)	57.8 (30.0–149.3)	63.7 [31.3–108.7]	4.3 × 10^–9^	**↑↑**
E-Selectin (ng/ml)	52.0 (32.7–65.5)	38.7 (27.7–49.3)	-12.6 [-18.5– -6.4]	6.7 × 10^–5^	↓
L-Selectin (ng/ml)	582.0 (404.9–878.8)	559.9 (346.6–703.0)	-92.3 [-161.5–28.5]	0.003	↔
P-Selectin (ng/ml)	35.8 (27.3–46.2)	47.3 (35.8–54.4)	9.6 [5.0–14.9]	1.9 × 10^–4^	**↑**
ICAM-1 (ng/ml)	276.9 (190.5–387.6)	272.9 (191.3–408.6)	7.8 [-22.8–37.7]	0.63	↔
VCAM-1 (ng/ml)	92.4 (63.7–132.9)	100.1 (78.3–152.3)	10.0 [-4.8–22.3]	0.15	↔

Adjusted α = 0.002. *CI* confidence interval, *CPB* cardiopulmonary bypass, *C* complement, *CF* complement factor, *CCL* CC chemokine ligand, *CPB* cardiopulmonary bypass, *CX Time* cross-clamp time, *CXCL* CXC chemokine ligand, *ET1* endothelin-1, *GM-CSF* granulocyte–macrophage colony-stimulating factor, *ICAM-1* intracellular adhesion molecule 1, *ICU LOS* intensive care unit length of stay, *IL* interleukin, *IQR* interquartile range, *OI* oxygenation index, *TNF* tumor necrosis factor, *TRAIL* tumor necrosis factor-related apoptosis-inducing ligand, *VCAM*-1vascular cell adhesion molecule 1

### Post-operative clinical outcomes and scores

**Fig. 2:**
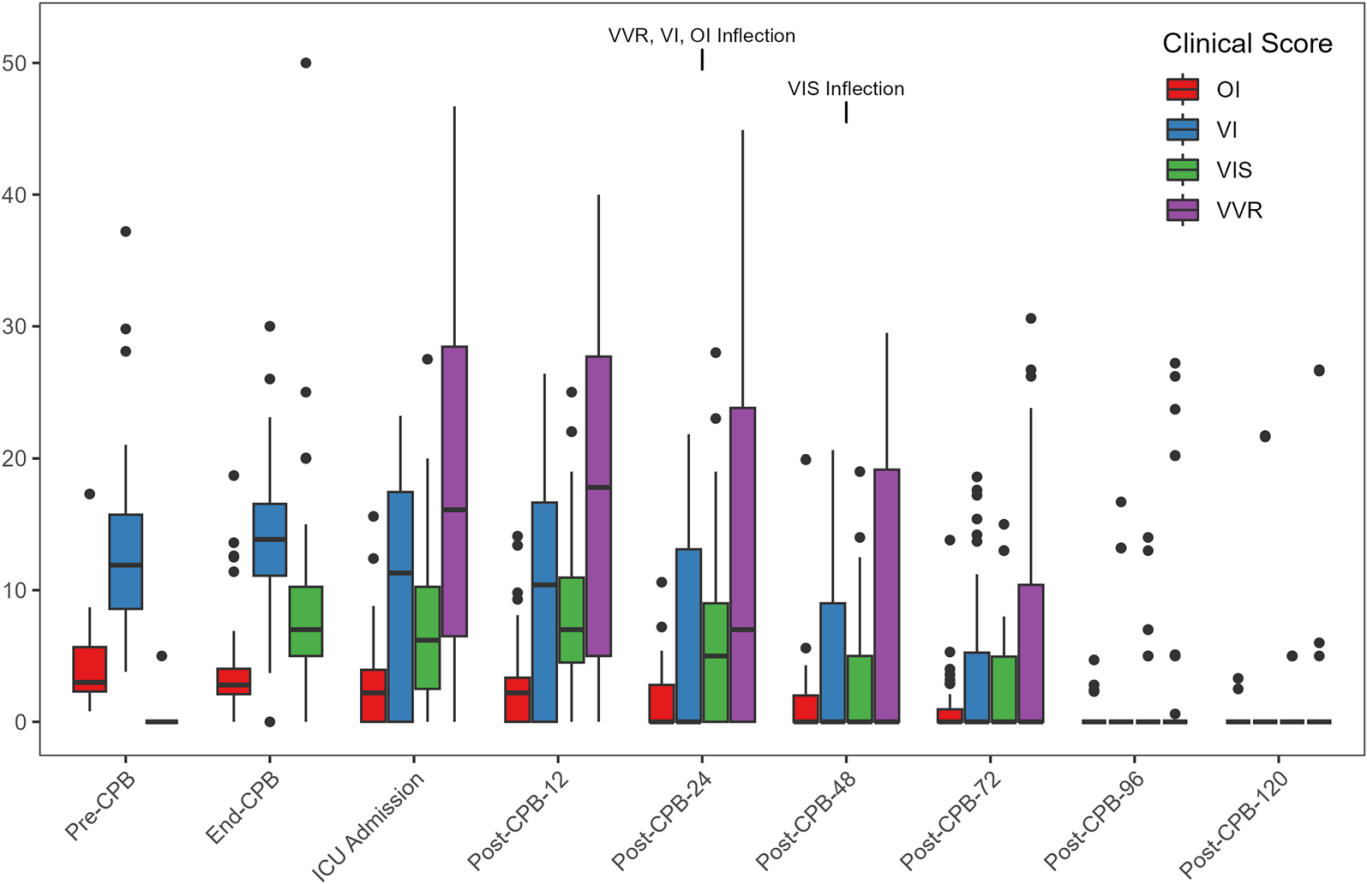
Time series of cardiopulmonary and vasomotor scores. Pre-CPB time point is the baseline with subsequent time points defined by hours “Post-CPB” cessation. VVR, VI, OI inflection and the VIS inflection indicate the timepoint of a stable downward trend for each clinical score. VVR was not calculated at Pre-CPB or End-CPB as renal function assessment was not conducted. The middle horizontal line of the boxplots represents the median while the lower and upper borders of the box represent the 25th percentile and 75th percentile. The lower and upper whiskers represent the minimum and maximum values of non-outliers while dots represent outliers. CPB: cardiopulmonary bypass; OI: oxygenation index; VI: ventilation index; VIS: vasoactive-inotrope score; VVR: vasoactive-ventilation-renal score

There were no 30-day mortalities, and one patient who required mechanical circulatory support was weaned and progressed to hospital discharge. Acute kidney injury was relatively uncommon; only 3 patients had a grade 1 injury which all resolved. The ventilation time was 19 (0–70) hours, and the standardized ICU LOS was 62 (24–95) hours. The individual peak clinical scores are summarized in Table 2, while the clinical score time series are depicted in Fig. 2. VVR scores were not statistically different between ICU admission and Post-CPB-12 h (MD = 0.2; *p* = 0.87) but steadily decreased relative to ICU admission at Post-CPB-24 h (MD = − 4.6; *p* = 0.004) and thereafter. VIS was substantially higher at End-CPB relative to Pre-CPB baseline (MD = 8.0; *p* = 3.69 × 10^–7^), unchanged between End-CPB and ICU admission (MD = − 1.0; *p* = 0.19), held steady relative to ICU admission at Post-CPB-12 h (MD = 1.0; *p* = 0.22) and Post-CPB-24 h (MD = − 2.0; *p* = 0.11) then began to decline at Post-CPB-48 h (MD = −5.0; *p* = 4.99 × 10^–4^). VI was unchanged between Pre-CPB and End-CPB (MD = 1.5; *p* = 0.15), decreased between End-CPB and ICU admission (MD = − 4.2; *p* = 0.003) due to 13 patients (33%) extubated in the operating room, then was stable between ICU admission and Post-CPB-12 h (MD = − 1.0; *p* = 0.40) and began to decline at Post-CPB-24 h (MD = − 5.8; *p* = 0.001) and thereafter. OI showed similar behavior to VI as it was unchanged between Pre-CPB and End-CPB (MD = − 0.2; *p* = 0.67), decreased between End-CPB and ICU admission (MD = − 1.5; *p* = 7.09 × 10^–4^) due to extubation in the operating room, then was stable between ICU admission and Post-CPB-12 h (MD = − 0.3; *p* = 0.54) then began to decline at Post-CPB-24 h (MD = − 1.3; *p* = 0.02) and after that. Overall, organ dysfunction, represented by elevated clinical scores, was most significant within the first 48 h, followed by a decreasing trend through the following post-operative days.

### Principal component analysis and hierarchical clustering on principal components

**Fig. 3:**
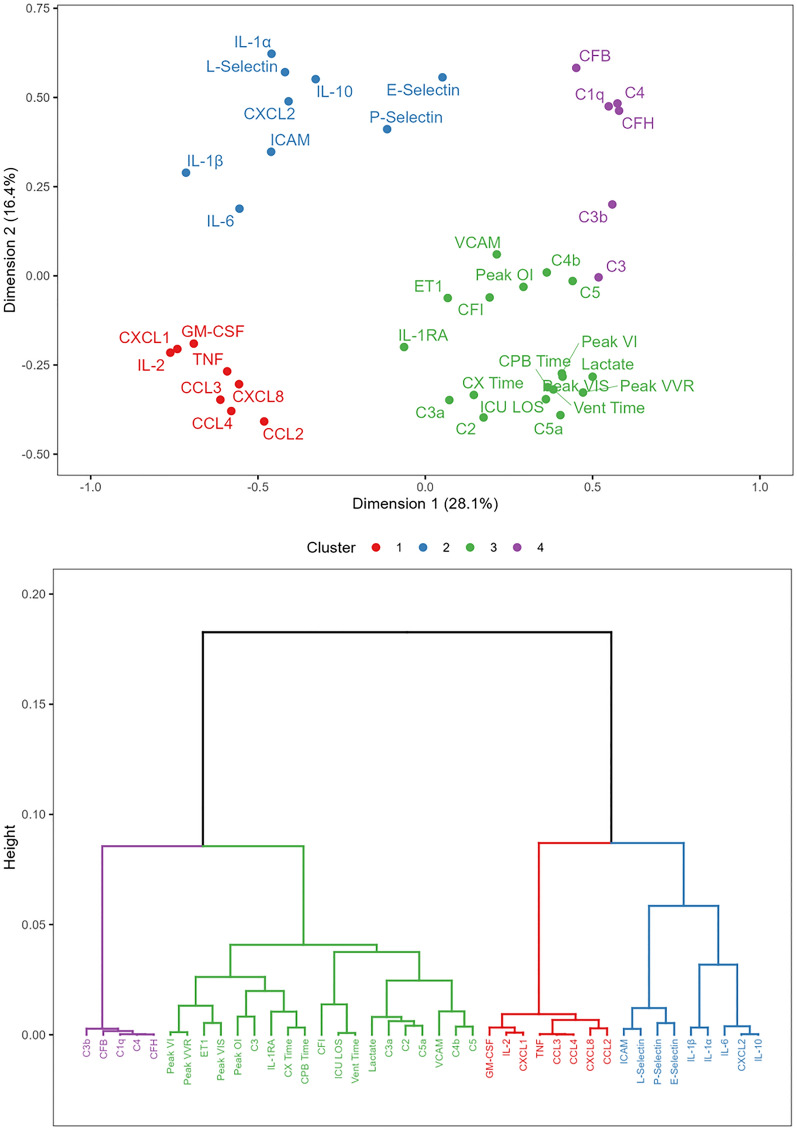
Principal component analysis and hierarchal clustering dendrogram. Mediator values reflect the concentrations at End-CPB. Cluster 3 is significantly different from clusters 1 (*p* = 0.004), 2 (*p* = 5.79 × 10^–4^) and 4 (*p* = 0.034). C: complement; CF: complement factor; CCL: CC chemokine ligand; CPB: cardiopulmonary bypass; CX Time: cross-clamp time; CXCL: CXC chemokine ligand; ET1: endothelin-1; GM-CSF: granulocyte–macrophage colony-stimulating factor; ICAM: intracellular adhesion molecule; ICU LOS: intensive care unit length of stay; IL: interleukin; OI: oxygenation index; TNF: tumor necrosis factor; TRAIL: tumor necrosis factor-related apoptosis-inducing ligand; VCAM: vascular cell adhesion molecule; Vent Time: ventilation time; VI: ventilation index; VIS: vasoactive-inotrope score; VVR: vasoactive-ventilation-renal score

The PCA revealed 11 dimensions that accounted for 85% of the variance in the dataset. The first dimension described 28.1% of the variance, and the second dimension explained 16.4% of the variance. The hierarchical clustering revealed 4 clusters depicted on dimensions one and two and a dendrogram in Fig. 3. Variables within the same cluster are the most alike and correlate with one-another and are more distinct from variables in other clusters. Cluster 1 contained pro-inflammatory cytokines—TNF, IL-2, and GM-CSF—and chemokines including CCL2, CCL3, CCL4,CXCL1 and CXCL8. Cluster 2 included pro-inflammatory cytokines—IL-1α, IL-1β, IL-6 and TRAIL—the anti-inflammatory mediator IL-10, the chemokine CXCL2, as well as the leukocyte adhesion molecules E-selectin, P-selectin, L-selectin and ICAM-1. Cluster 3 contained all clinical variables—lactate, CPB time, cross-clamp time, peak VVR, peak VIS, peak VI, peak OI, ventilation time, ICU LOS—and the complement anaphylatoxins C3a and C5a, the complement factors C2, C4b, C5 and CFI, along with endothelin-1, the anti-inflammatory mediator IL-1Ra and the leukocyte adhesion molecule VCAM-1. Cluster 4 contained the activated complement factor C3b along with other complement proteins—C1q, C3, C4—and complement system regulators CFB and CFH. Mahalanobis distances are summarized in Table 4 and revealed that cluster 3 was significantly different from cluster 1, 2, and 4 through all 11 dimensions of the analysis. Notably, the clinical variables and complement anaphylatoxins in cluster 3 were distinct from pro-inflammatory cytokine and chemokine mediators in cluster 2 (Mahalanobis distance = 17.4; *p* = 5.79 × 10^–4^) and cluster 1 (Mahalanobis distance = 16.5; *p* = 0.004). Cluster 4 contained complement factor and was more closely related to cluster 3 (Mahalanobis distance = 13.6; *p* = 0.034) relative to clusters 1 and 2.

**Table 4:** Mahalanobis distance between clusters (*n* = 40)

Cluster comparison	Mahalanobis distance	Hotelling T^2^ statistic	F statistic	p-value
1 & 2	12.7	52.6	1.9	0.22
1 & 3	16.5	91.6	4.9	0.004
1 & 4	8.8	30.3	0.46	0.84
2 & 3	17.4	111.6	6.2	5.79 × 10^–4^
2 & 4	14.7	55.0	1.4	0.39
3 & 4	13.6	61.3	3.0	0.034

### Linear correlation

**Fig. 4:**
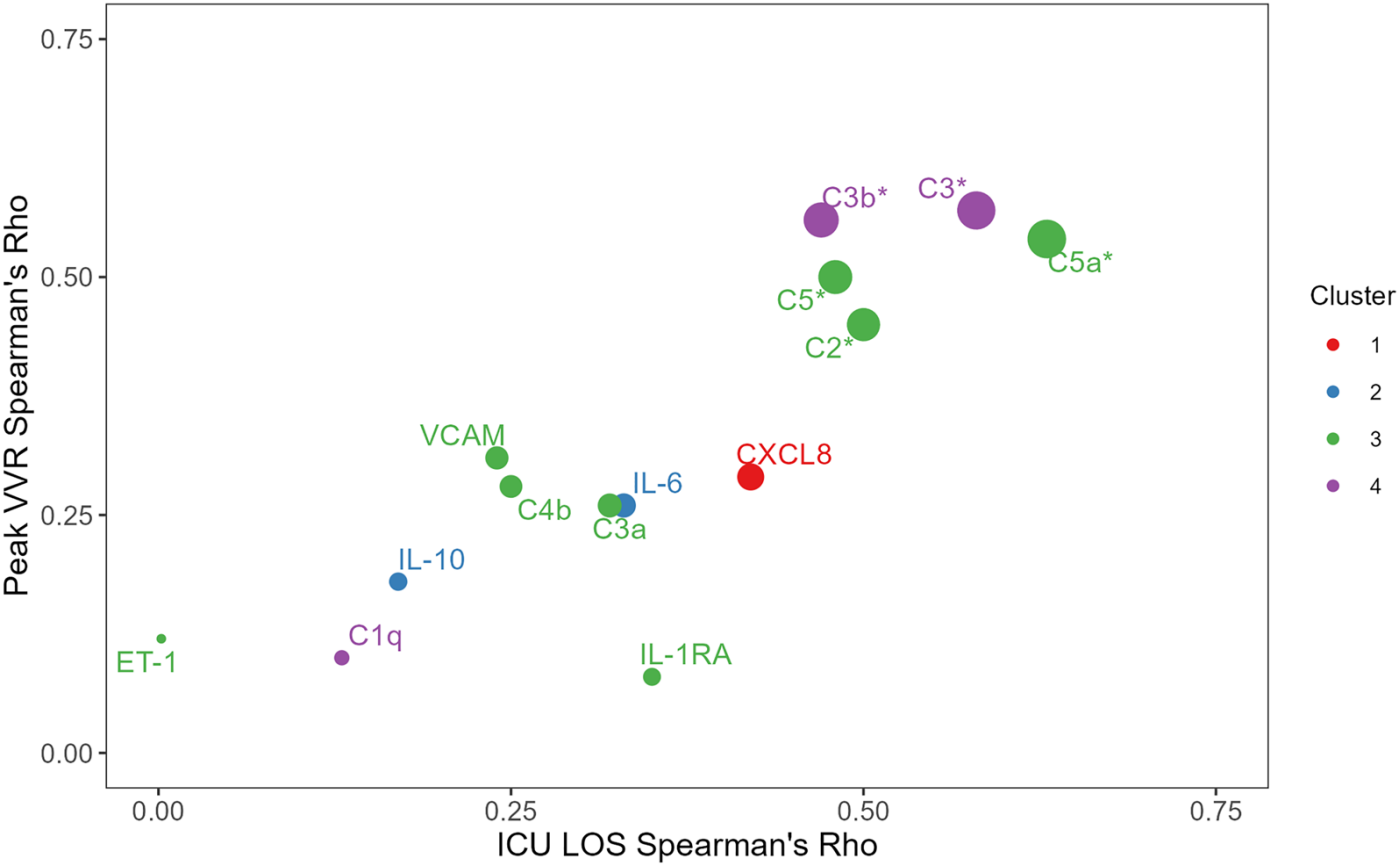
Linear correlation between End-CPB mediatorconcentration and post-operative critical care requirements. * denotes statistical significance with adjusted α = 0.004. *C* complement, *CXCL* *CXC* chemokine ligand, *ET1* endothelin-1, *ICU LOS* intensive care unit length of stay, *IL* interleukin, *VCAM-1* vascular cell adhesion molecule 1, *VVR* vasoactive-ventilation-renal score

C3b, C5a, IL-6, IL-10 and CXCL8 showed dynamic increases throughout CPB with over 5 times median fold change from baseline. Furthermore, C3a, C5a, C2, C4b, C5, ET-1, IL-1Ra and VCAM-1 were found in cluster 3 alongside post-operative clinical scores and outcomes. C3 was included in the correlation analysis because of its central biologic role in the complement system and close relation to cluster 3. The linear correlation between these mediator concentrations at the end of CPB with ICU LOS and peak VVR can be seen in Table 5 and depicted in Fig. 4. C2, C3, C3b, C5, and C5a showed significant correlation with increased critical care requirements while the other mediators did not. C5a concentrations at the end of CPB were significantly correlated with CPB time (Rho = 0.48, *p* = 0.002) while C3a was not (Rho = 0.21, *p* = 0.19). Finally, there was a strong correlation between peak VVR and ICU LOS with Rho = 0.73 (*p* = 1.70 × 10^–7^).

**Table 5:** Linear association between End-CPB [mediator] with peak VVR and ICU LOS (*n* = 40)

End-CPB [mediator]	Mediator cluster	Spearman’s Rho			
		Peak VVR	*p*	ICU LOS	*p*
C1q	4	0.10	0.24	0.13	0.42
C2	3	0.45	0.004	0.50	0.001
C3	4	0.57	1.80 × 10^–4^	0.58	1.10 × 10^–4^
C3a	3	0.26	0.11	0.32	0.045
C3b	4	0.56	2.30 × 10^–4^	0.47	0.002
C4b	3	0.28	0.09	0.25	0.13
C5	3	0.50	0.001	0.48	0.002
C5a	3	0.54	3.40 × 10^–4^	0.63	1.70 × 10^–5^
IL-6	2	0.26	0.11	0.33	0.037
IL-10	2	0.18	0.27	0.17	0.31
IL-1Ra	3	0.08	0.63	0.35	0.030
CXCL8	1	0.29	0.08	0.42	0.008
ET-1	3	0.12	0.45	0.00	1.00
VCAM-1	3	0.31	0.06	0.24	0.14

Adjusted α = 0.004. *C* complement, *CPB* cardiopulmonary bypass, *ET-1* endothelin-1, *ICU LOS* intensive care unit length of stay, *IL* interleukin, *VVR* vasoactive-ventilation-renal score, *VCAM-1* vascular cell adhesion molecule 1

## Discussion

We conducted an exploratory analysis across a wide range of inflammatory mediators to identify those related to adverse and prolonged post-operative outcomes following pediatric heart surgery. Our results included the assessment of 33 inflammatory mediators throughout CPB—from complement, cytokine, chemokine and leukocyte adhesion pathways—which is the most comprehensive to date. We observed dynamic increases of C3a, C5a, IL-6, IL-1Ra, IL-10,CCL2 and CXCL8 while IL-1α, IL-1β and TNF were largely static or decreased at the end of CPB relative to baseline. There was clear evidence of complement activation during CPB with markedly elevated C3, C3a, C3b and C5a. Importantly, these dynamic increases contrast to classical pathway complement molecules C1q, C2, C4, CFB, CFH and CFI concentrations which were largely unchanged throughout the CPB time. Therefore, it is unlikely that the alternative complement factor changes (C3, C3a, C3b, C5 and C5a) are explained by hemoconcentration [10]. Interestingly, there was a marked discrepancy between C3 cleavage product quantities as C3a (1.8× median fold increase from baseline) was far less prevalent than C3b (39.3x median fold increase from baseline) at the end of CPB. The metabolism of C3a and C3b are known to differ. C3a is preferentially degraded with a half-life of 30 min, while C3b goes onto form the C3 convertase machinery which is stabilized by regulator proteins [23]. Further, this observation could also be explained by the effective extraction of C3a by ultrafiltration (sieving coefficient = 1019%), as C3b is too large to be removed through the membrane pores (sieving coefficient = 0%) [10].

Two statistical methods were used to explore the relationship between mediator concentrations at the end of CPB and post-operative clinical scores and outcomes. First, a PCA-HCPC revealed that complement factors and the anaphylatoxins C3a and C5a are closely related to the CPB exposure, myocardial ischemia time during aortic cross-clamp and subsequent clinical instability depicted by peak VVR, -VIS, -VI and -OI along with ventilation time and ICU LOS. Second, linear correlation analyses between purposefully selected mediators with both ICU LOS and peak VVR identified C2, C3, C3b, C5 and C5a as most associated with adverse post-operative recovery. Unexpectedly, C3a was not found to be linearly correlated to post-operative critical care requirements or CPB time. C3a is known to have a substantially higher ultrafiltration sieving coefficient (1019%) than C5a (49%), thereby, the therapy could attenuate the circulating C3a concentrations and magnitude of physiologic impact relative to C5a [10]. Furthermore, C5a is 20 times more biologically potent than C3a which might also explain the more robust relationship between C5a and post-operative critical illness [24]. Remarkably, the analysis failed to identify any classic pro-inflammatory cytokines TNF, IL-1α, IL-1β, IL-6 or CXCL8 as mediators relevant to post-operative recovery.

Complement activation by CPB was first identified in the 1980s by James Kirklin [25]. Since then, elevated levels of pro-inflammatory mediators such as C3a, C5a, TNF, IL-1, IL-6, CXCL8 and anti-inflammatory mediators IL-1Ra and IL-10 have been routinely observed during CPB exposure [2, 4]. C3 is cleaved into C3a and C3b during alternative complement activation and common pathway propagation [26]. C3a and C5a are potent anaphylatoxins that induce endothelial dysfunction and neutrophil activation, known as an effector function of complement, while C3b is a key subunit in the C3 convertase required for complement propagation [26, 27]. IL-1α functions as an “alarmin” as the active form is released from injured and dying cells, while IL-1β requires intra- or extra-cellular cleavage to become activated and elicit innate immune mechanisms [28]. TNF is a hallmark of systemic inflammation and is produced by several immunologic cells and endothelium when stimulated by IL-1, C5a, and other substances [29]. Our results generate two interesting observations for future investigations. First, there was no meaningful change of IL-1α or IL-1β or TNF throughout CPB with SBUF–SMUF, but it is uncertain if extraction by ultrafiltration alone explains this finding. Both IL-1β and TNF are known to be extracted by ultrafiltration with sieving coefficients of 75% and 11%, respectively [10]. Second, many patients showed signs of clinically significant inflammation and cardiopulmonary dysfunction in the post-operative phase despite having unchanged concentrations of these three classic pro-inflammatory mediators. This suggests that complement activation and effector functions alone could potentially be sufficient to illicit the observed clinical inflammatory sequelae.

Despite the common cognitive assumption by clinicians that the entire innate immune response is causal to post-operative outcomes after children’s heart surgery, there is in fact a paucity of scientific evidence to confirm it. Kirklin et al*.* identified C3a burden as a risk factor for post-operative cardiac, pulmonary and renal dysfunction [25]. Seghaye et al*.* concluded that complement activation, denoted by C3 conversion, but not C5a was associated with multi-organ failure following children’s heart surgery [30]. Allan et al*.* showed that IL-6 and CXCL8 concentrations measured immediately after CPB have a weak linear association with ICU LOS with a Spearman Rho = 0.29 (*p* = 0.06) and Rho = 0.30 (*p* = 0.004), respectively [31]. Building upon knowledge to date, our novel and comprehensive analysis suggests that complement factors, rather than pro-inflammatory cytokines and chemokines, are crucial mediators associated with clinical outcome parameters. Therefore, we hypothesize that the inhibition of C3 activation, C3 convertase feed-forward propagation or the sequestration of C3a and C5a should be considered as potential immunomodulatory therapies to dampen CPB-associated inflammation to improve post-operative clinical outcomes. Specifically, continuous ultrafiltration could potentially have a clinically significant anti-inflammatory effect by sieving off C3a and C5a, although further comparative research is required.

The authors recognize limitations in this investigation. First, the patient population is heterogenous in several important variables that could potentially modulate a patient’s immunologic response to CPB and post-operative recovery: age, presence of cyanotic congenital heart disease, single ventricular physiology, deep hypothermic circulatory arrest, CPB sanguineous prime and use of prophylactic steroids. The small sample size is prohibitive to analytic restriction which might focus on a more homogenous patient group to control for selected confounding variables. Second, the data are drawn from a single-center sample which impacts generalizability, and the results should be interpreted in the context of pediatric CPB and continuous SBUF . The immunologic signature and corresponding clinical courses of these patients could differ if continuous ultrafiltration was not used, hypothetically, the inflammatory burden would be greater and correspond with more organ dysfunction post-operatively. Third, despite this study investigating the largest number of inflammatory mediators during CPB to date, there is a possibility of unmeasured mediators that are vital to CPB-associated inflammation but not here evaluated. Furthermore, mediator concentrations are measured in the circulation and might differ from that in the capillaries and tissues. Fourth, the exploratory principal component analysis results cannot demonstrate causality, but serves as a robust platform for future research.

## Conclusion

Pediatric cardiac surgery and CPB elicits a systemic inflammatory response characterized by complement system activation and production of the pro-inflammatory cytokine IL-6, the chemokine CXCL8, and the anti-inflammatory mediators IL-1Ra and IL-10. The burden of activated complement mediators represented by circulating C3, C3a, C3b, C5 and C5a are most related to post-operative cardiopulmonary dysfunction and prolonged critical care requirements as the patient recovers from the surgical and inflammatory insult. Despite the dynamic production of pro-inflammatory cytokines, the concentrations of these mediators were not found to be related to post-operative morbidity or recovery, in notable contrast to the complement factors. Our resultssuggest that future innovation should focus on therapies that inhibit complement activation, propagation, and effector functions to enhance recovery for infants and children undergoing congenital cardiac surgery.

## Supplementary Information

Additional file 1.

## Data Availability

The datasets generated and analyzed are not publicly accessible due to patient data and information confidentiality but could be available from the corresponding author on reasonable request.

## Abbreviations

C: Complement
CF: Complement factor
CI: Confidence interval
CCL: C–C motif chemokine ligand
CXCL: C-X-C motif chemokine ligand
CPB: Cardiopulmonary bypass
DHCA: Deep hypothermic circulatory arrest
ET1: Endothelin-1
GM-CSF: Granulocyte–macrophage colony-stimulating factor
ICAM-1: Intercellular adhesion molecule 1
IL: Interleukin
MD: Median difference
OI: Oxygenation index
SBUF: Subzero-balance ultrafiltration
SMUF: Simple modified ultrafiltration
STAT: Society of Thoracic Surgeons-European Association for Cardio-Thoracic Surgery
TNF: Tumor necrosis factor
TRAIL: Tumor necrosis factor-related apoptosis-inducing ligand
VCAM-1: Vascular cell adhesion molecule 1
VI: Ventilation index
VIS: Vasoactive-inotrope score
VVR: Vasoactive-ventilation-renal score
