# Supplementary material for: Unmasking culprits: novel analysis identifies complement factors as potential therapeutic targets to mitigate inflammation during children's heart surgery
Source: Eur J Med Res. 2024 Dec 19;29:601. doi: 10.1186/s40001-024-02156-0 (PMC11657506; doi:10.1186/s40001-024-02156-0)
Supplement: Supplementary file 1 — Additional file 1. [file 40001_2024_2156_MOESM1_ESM.pdf]

PICU Discharge Checklist
Admit Time:
Completion Time:
*Completion Time is recorded as the time when the final criteria is met.

CNS	☐ Stable neurological status.<br>☐ No concern about persistent, new, evolving or worsening neurological injury.<br>☐ Off sedative infusion for at least for 6 hours.<br>☐ On stable or decreasing IV opioid infusion.
CVS	☐ Improving or stable hemodynamics and perfusion for at least 12 hr.<br>☐ Stable Rhythm.<br>☐ Off all cardio active infusions for at least 6 hr.<br>☐ In case of a chronic heart failure, on a stable infusion of milrinone @ $\leq$ 0.5 mcg/kg/min. and not requiring continuous intensive care monitoring.<br>☐ No concern of persistent, new, evolving or worsening cardiopulmonary function or pathology.<br>☐ No planned intervention over next 24 hr needing close cardiorespiratory monitoring.<br>☐ Off arterial line.<br>☐ Stable on temporary pacemaker.
Respiratory	☐ At least 12-hour post extubation.<br>☐ Stable respiratory status.<br>☐ No concern of persistent, new, evolving or worsening lung pathology.<br>☐ Saturations in acceptable range as per underlying CHD.<br>☐ Respiratory rate within the normal range l for the age.<br>☐ No work of breathing/respiratory distress.<br>☐ On decreasing or stable minimal respiratory support.<br>☐ Low flow oxygen (0.5 -3 lt/min) via nasal prongs.<br>☐ On Stable (Max flow 2 lt/kg) or decreasing high flow nasal settings with a plan of long term wean.
GI	☐ Stable GI status.<br>☐ Tolerating feeds or at least clear fluids.<br>☐ No need of close monitoring while advancing feeds.<br>☐ No concern of persistent, new, evolving or worsening GI pathology.
GU	☐ Acceptable fluid status and urine output (> 0.5 ml/kg) with/out diuretics.<br>☐ Not on Continuous infusion of diuretics.<br>☐ No concern of persistent, new, evolving or worsening renal function as indicated by clinical and/or laboratory parameters.
ID	☐ No concern of new, evolving, or worsening infection.
Hematology	☐ On stable anticoagulation.<br>☐ Not on any anticoagulation medication which requires frequent lab and closed clinical monitoring.
Acid Base and Electrolytes	☐ Stable electrolytes parameters with/out supplementation.<br>☐ Not requiring continuous intravenous infusion of electrolytes.<br>☐ Acid base status in stable range.<br>☐ No concern of persistent or new or evolving or worsening acid base status.
	☐ Discharged by PICU Attending.
